# NAMPT inhibition sensitizes pancreatic adenocarcinoma cells to tumor-selective, PAR-independent metabolic catastrophe and cell death induced by *β*-lapachone

**DOI:** 10.1038/cddis.2014.564

**Published:** 2015-01-15

**Authors:** Z Moore, G Chakrabarti, X Luo, A Ali, Z Hu, F J Fattah, R Vemireddy, R J DeBerardinis, R A Brekken, D A Boothman

**Affiliations:** 1 Pharmacology and Radiation Oncology, Simmons Comprehensive Cancer Center, University of Texas Southwestern Medical Center, Dallas, TX, USA; 2 Internal Medicine and Touchstone Diabetes Center, Simmons Comprehensive Cancer Center, University of Texas Southwestern Medical Center, Dallas, TX, USA; 3 Children's Medical Center Research Institute, Simmons Comprehensive Cancer Center, University of Texas Southwestern Medical Center, Dallas, TX, USA; 4 Surgical Oncology, Department of Surgery and Hamon Center for Therapeutic Oncology Research, Simmons Comprehensive Cancer Center, University of Texas Southwestern Medical Center, Dallas, TX, USA

## Abstract

Nicotinamide phosphoribosyltransferase (NAMPT) inhibitors (e.g., FK866) target the most active pathway of NAD^+^ synthesis in tumor cells, but lack tumor-selectivity for use as a single agent. Reducing NAD^+^ pools by inhibiting NAMPT primed pancreatic ductal adenocarcinoma (PDA) cells for poly(ADP ribose) polymerase (PARP1)-dependent cell death induced by the targeted cancer therapeutic, *β*-lapachone (*β*-lap, ARQ761), independent of poly(ADP ribose) (PAR) accumulation. *β*-Lap is bioactivated by NADPH:quinone oxidoreductase 1 (NQO1) in a futile redox cycle that consumes oxygen and generates high levels of reactive oxygen species (ROS) that cause extensive DNA damage and rapid PARP1-mediated NAD^+^ consumption. Synergy with FK866+*β*-lap was tumor-selective, only occurring in NQO1-overexpressing cancer cells, which is noted in a majority (∼85%) of PDA cases. This treatment strategy simultaneously decreases NAD^+^ synthesis while increasing NAD^+^ consumption, reducing required doses and treatment times for both drugs and increasing potency. These complementary mechanisms caused profound NAD(P)^+^ depletion and inhibited glycolysis, driving down adenosine triphosphate levels and preventing recovery normally observed with either agent alone. Cancer cells died through an ROS-induced, *μ*-calpain-mediated programmed cell death process that kills independent of caspase activation and is not driven by PAR accumulation, which we call NAD^+^-Keresis. Non-overlapping specificities of FK866 for PDA tumors that rely heavily on NAMPT-catalyzed NAD^+^ synthesis and *β*-lap for cancer cells with elevated NQO1 levels affords high tumor-selectivity. The concept of reducing NAD^+^ pools in cancer cells to sensitize them to ROS-mediated cell death by *β*-lap is a novel strategy with potential application for pancreatic and other types of NQO1+ solid tumors.

An emerging metabolic target for the treatment of recalcitrant cancers, such as pancreatic adenocarcinoma (PDA), is their reliance on NAD^+^ synthesis, particularly through the nicotinamide-recycling pathway.^1, 2, 3^ Rapid and efficient NAD^+^ synthesis is critical to sustain signaling processes, such as deacetylation by sirtuins and adenosine diphosphate (ADP) ribosylation by poly(ADP ribose) polymerase 1 (PARP1). NAD(P)^+^ pools are also necessary to support anabolic metabolism and proliferation of cancer cells. In an attempt to leverage increased tumor-cell reliance on NAD^+^ synthesis, small molecule inhibitors of nicotinamide phosphoribosyltransferase (NAMPT) were developed (e.g., FK866).^4^ NAMPT catalyzes the rate-limiting step of the most active pathway of NAD^+^ synthesis. Inhibitors of NAMPT, such as FK866, reduce NAD^+^ levels, induce canonical apoptosis preferentially in cancer cells *in vitro*, inhibit tumor growth, and increase overall survival in preclinical cancer models.^1, 5, 6, 7^ FK866 (APO866) was relatively well tolerated in humans and advanced to phase II clinical trials. However, owing to its short half-life in circulation, prolonged treatment regimens were required and toxicity to normal, rapidly proliferating hematopoietic cells was noted. Accordingly, FK866 and other NAMPT inhibitors did not demonstrate sufficient tumor-selectivity to achieve clinical success as single agents.^8^

To increase the specificity and efficacy of NAMPT inhibition, we combined FK866 with *β*-lapachone (*β*-lap), a targeted cancer therapeutic that causes tumor-selective PARP1 hyperactivation and NAD^+^ depletion in an NADPH:quinone oxidoreductase 1 (NQO1)-specific manner.^9^ *β*-Lap is a substrate for two-electron oxidoreduction by NQO1, a Phase II quinone-detoxifying enzyme.^9^ The resulting hydroquinone form of *β*-lap is highly unstable and spontaneously reacts with oxygen to revert back to the parent compound, generating two moles of superoxide per mole of NAD(P)H used in the process. This results in a futile cycle that occurs rapidly in NQO1-overexpressing cells, causing marked NADH/NADPH oxidation. DNA damage in the form of base oxidation and DNA single-strand breaks results from H_2_O_2_ generated from the futile redox cycle. In an attempt to repair this damage, PARP1 becomes hyperactivated, generating extensive branched poly(ADP ribose) (PAR) polymer. Hyperactivated PARP1 substantially depletes NAD^+^ and ultimately adenosine triphosphate (ATP) levels, thereby inhibiting subsequent repair of *β*-lap-induced DNA lesions. The observed cell death is caspase-independent and driven by nuclear translocation of apoptosis-inducing factor (AIF), activation of *μ*-calpain, and post-translational modification of GAPDH.^10, 11, 12, 13^ NQO1 is highly expressed in many types of cancer, and the therapeutic window provided by NQO1 bioactivation of *β*-lap has advanced its use to phase I clinical trials (ARQ761).^14^ Elevated NQO1 expression (≥10-fold) has been identified in ~85% of patient tissue from pancreatic ductal adenocarcinoma (PDA), making pancreatic cancer an especially appealing target for therapy using NQO1 bioactivatable drugs, such as *β*-lap.^15, 16, 17, 18^ However, dose-limiting methemoglobinemia caused by nonspecific reactive oxygen species (ROS) generation at high *β*-lap doses may limit the efficacy of *β*-lap as monotherapy.^19^ Strategies for increasing cancer cell cytotoxicity while maintaining NQO1 specificity could enhance use of *β*-lap for therapy against PDAs, as well as other solid cancers that overexpress NQO1.

We found that examining cell death pathways induced by *β*-lap, with or without FK866 treatment, is a novel means to elucidate general mechanisms of lethality mediated by NAD^+^ loss, as cell death by PARP1 hyperactivation occurs in other contexts. Notably, cell death induced by ischemia/reperfusion shares many of the same characteristics: ROS induction, PARP1 hyperactivation, calcium release, AIF translocation, and caspase-independence.^20, 21^ Similarly, treatment with methylnitronitrosoguanidine (MNNG; a DNA alkylating agent) or induction of neuronal excitotoxicty induces PARP1 hyperactivation and cell death, but without futile cycle-induced ROS production.^22, 23, 24^ Recent studies suggest an important role for accumulated free PAR polymer that can directly activate *μ*-calpain, activate and release AIF, and inhibit glycolysis.^22, 25, 26, 27, 28^ By combining *β*-lap and FK866, we uncouple NAD^+^ and ATP depletion from the robust formation of PAR noted with *β*-lap alone, allowing us to define the function of PAR formation in *β*-lap-induced cell death.

*β*-Lap and FK866 have distinct, but highly complementary mechanisms of action. *β*-Lap induces tumor-selective NAD^+^ depletion specifically in cancer cells that express high levels of NQO1. FK866 primes cancer cells for cell death by lowering NAD^+^/NADH pools and prevents recovery by inhibiting NAD^+^ synthesis from nicotinamide liberated by activated PARP1. We show that the increased dependence of PDA cells on glycolysis is specifically targeted by ROS-induced, NAD^+^ depletion caused by exposure to both drugs. Glycolytic inhibition, ATP depletion, and cell death is independent of PAR formation, strongly suggesting that PAR accumulation is not directly involved. The use of *β*-lap with NAMPT inhibitors results in synergistic NQO1- and PARP1-dependent cancer cell death, allowing the use of lower doses and shorter treatment times for both therapeutics.

## Results

### FK866 pretreatment sensitizes PDA cells to *β*-lap

**Figure 1:**
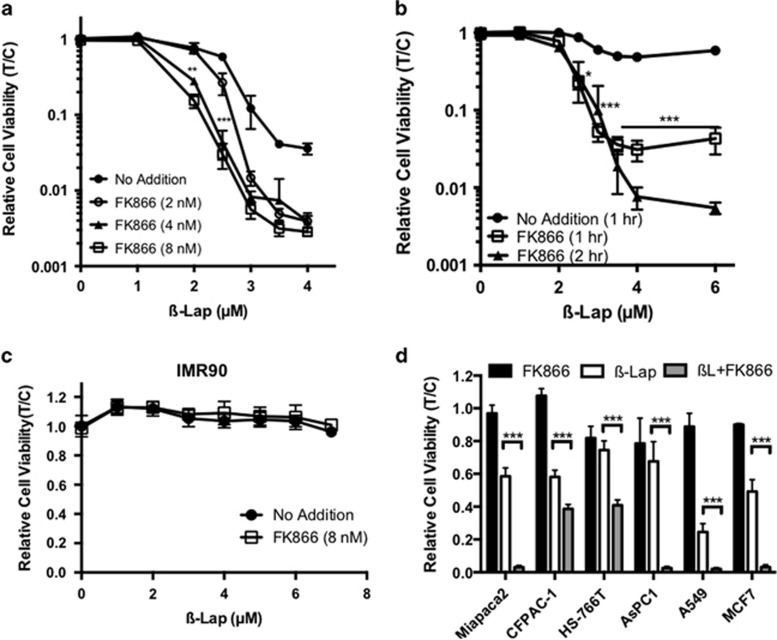
FK866 pre- and co-treatment optimally sensitizes PDA cells to *β*-lap. (**a**) MiaPaca2 cells were pretreated for 24 h with FK866 or vehicle alone at indicated doses. Cells were washed and then exposed to *β*-lap for 2 h, with or without FK866, as indicated. Relative cell viability (T/C) was monitored by CellTiter-Glo 24 h later. All luminescence values were normalized to untreated cells (no FK866 or *β*-lap). (**b**) FK866 pre- and co-treatment greatly reduces the minimum effective treatment time for *β*-lap. MiaPaca2 cells were treated as in **a** with 8 nM FK866 and then exposed to *β*-lap for 1 or 2 h in the presence or absence (no addition) of 8 nM FK866. Drugs were removed and relative cell viability (T/C) was assessed 24 h later. (**c**) Normal human embryonic IMR-90 fibroblasts were treated with FK866 and *β*-lap as in **a** and relative cell viability was assessed. (**d**) Additional NQO1+ PDA cell lines (CFPAC-1, HS766T, and AsPC1), as well as breast (MCF7) and non-small cell lung (A549) cancer cells were treated with FK866 alone, *β*-lap alone, or with the combination as in **a** and assessed for relative cell viability (T/C) as in **a**. All results were compared using Student's *t*-tests (*n*=3 with mean ± S.D.) as indicated. *P<0.05;**P<0.01; ***P<0.001

We hypothesized that pretreatment of NQO1-overexpressing PDA cells with FK866 would sensitize them to subsequent *β*-lap exposure by lowering the available NAD^+^ pools, thereby increasing the ability of PARP1 to deplete the remaining NAD^+^ when stimulated by NQO1-induced ROS generation. MiaPaca2 cells were pretreated with FK866 for 24 h, then exposed to *β*-lap+FK866 for 2 h. Pre-treatment with FK866 resulted in increased sensitivity to *β*-lap, at normally sublethal and higher doses of the drug (Figure 1a). At LD_90_ levels of *β*-lap, FK866 pretreatment led to a dose enhancement ratio of 1.6, which was saturated by 4 nM FK866 (*P*=0.0018). Whereas FK866 is lethal to MiaPaca2 cells at low nanomolar concentrations with a long-term 72 h treatment (Supplementary Figure S1A), a short-term (24 h+2 h) treatment had no effect on viability (Figure 1a, all results normalized to untreated cells). Clonogenic survival was also decreased in MiaPaca2 cells exposed to FK866+*β*-lap *versus* *β*-lap treatment alone (*P*=0.0004) (Supplementary Figure S1B).

We optimized the ability of FK866 to sensitize PDA cells to *β*-lap, examining dose–response and time-course exposures. We previously determined that the minimum exposure time to induce cell death for *β*-lap was ~2 h with LD_50_ values of ~3 *μ*M in most cells, where NQO1+ cells are eliminated regardless of cell cycle position or p53 status.^29^ Interestingly, the minimum *β*-lap treatment time required to induce cell death was decreased to 1 h with FK866 and *β*-lap combination treatment (Figure 1b). In contrast, normal human IMR-90 embryonic lung fibroblasts that express low levels of NQO1 remained resistant to *β*-lap, with or without FK866 pretreatment (Figure 1c), suggesting that toxicity to normal cells was not increased by FK866 pretreatment.^9^ As with MiaPaca2 cells, the survival of other PDA cells showed similar hypersensitivity to *β*-lap+FK866 compared with either drug alone (Figure 1d). Furthermore, other NQO1+ overexpressing cancer cells, such as A549 non-small cell lung cancer cells, showed synergistic lethality with this combination treatment.

### FK866+*β*-lap synergy is NAMPT- and NQO1-dependent

**Figure 2:**
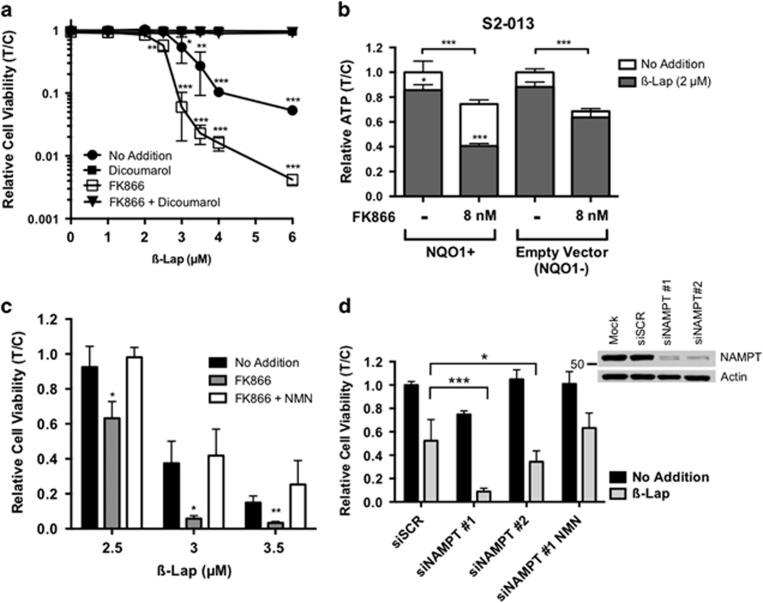
Synergy between FK866 and *β*-lap is NQO1-dependent and NAMPT-specific. (**a**) MiaPaca2 cells were pretreated with or without FK866 (8 nM), washed, and then exposed to various doses of *β*-lap (*μ*M) with or without 8 nM FK866 in the presence or absence of dicoumarol (50 *μ*M) for 2 h. Viability assays were then used to monitor survival 24 h after treatment. (**b**) S2-013 (NQO1-) PDA cells were corrected for NQO1 expression by CMV-NQO1 stable transfection as described.^30^ Cells were treated as in **a** and assessed for relative ATP content (T/C) 2 h later. (**c**) Nicotinamide mononucleotide (NMN, 500 *μ*M), which bypasses the NAMPT-catalyzed reaction to rescue NAMPT inhibition, was added during FK866 pretreatment and *β*-lap exposure as in **a**. NMN prevented sensitization by FK866, but did not spare *β*-lap-induced lethality. (**d**) Knockdown of NAMPT increased sensitivity to *β*-lap. Two unique siRNAs specific for the coding region of NAMPT were used to suppress protein expression of NAMPT (inset). Cells were then treated with *β*-lap (3 *μ*M, 2 h) and a viability assay was performed 24 h later. Results were compared using Student's *t*-tests (*n*=3 with mean ± S.D.), *P<0.05; **P<0.01; ***P<0.001

A series of small molecule and genetic experiments were used to define the target specificity of FK866+*β*-lap combination therapy. Dicoumarol (Dic), a small molecule inhibitor of NQO1, completely prevented the cytotoxicity of MiaPaca2 cells treated with *β*-lap alone or FK866+*β*-lap (Figure 2a). As Dic is an NADH mimic and may have off-target effects, we developed genetically matched NQO1+ and NQO1− PDA cells from parental NQO1 polymorphic NQO1- S2-013 cells as described.^30^ Note that ATP depletion caused by FK866 alone was not dependent on NQO1 expression, whereas *β*-lap-induced cytotoxicity and synergy only occurred in NQO1+, and not in NQO1-, S2-013 cells (Figure 2b) (*P*=0.00012). In contrast, overexpression of NQO1 in IMR-90 resulted in increased *β*-lap sensitivity but no synergy with FK866, likely due to a decreased reliance on NAMPT-mediated NAD^+^ synthesis in these normal fibroblasts (Supplementary Figure S1C). Similar synergistic dose–response effects were observed with GMX1778, another NAMPT inhibitor (Supplementary Figure S1D).^31^ NAMPT specificity was demonstrated by adding nicotinamide mononucleotide (NMN, 500 *μ*M), the product of the NAMPT-catalyzed reaction, which rescued FK866-dependent hypersensitivity to *β*-lap (Figure 2c). Notably, NMN addition did not rescue combination cytotoxicity above that induced by *β*-lap alone (Figure 2c). Furthermore, the depletion of NAMPT mRNA and protein levels using specific siRNAs in MiaPaca2 cells (Figure 2d, inset) increased sensitivity to *β*-lap, and this hypersensitivity was rescued to basal *β*-lap sensitivity levels with NMN (Figure 2d). Thus, NAMPT inhibition and NQO1-dependent bioactivation of *β*-lap were necessary and sufficient for the observed synergistic lethality.

### FK866 pretreatment enhances energy depletion and prevents recovery from *β*-lap

**Figure 3:**
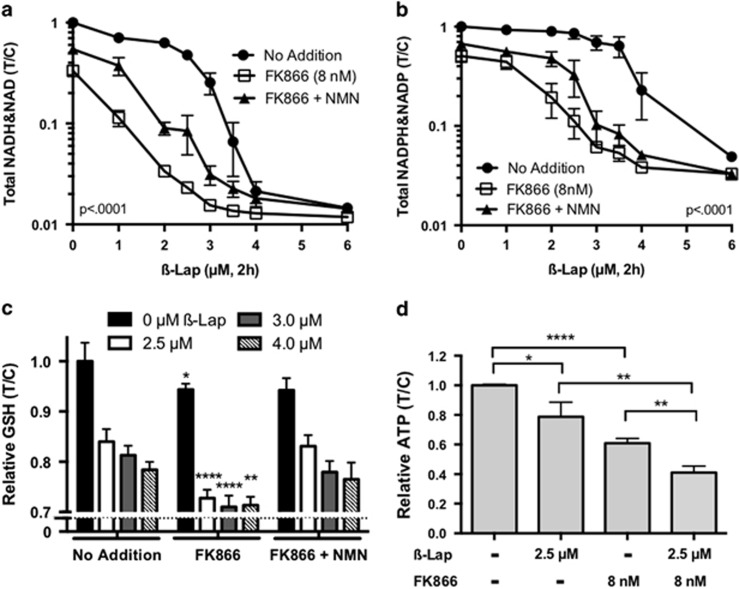
Exposure of MiaPaca2 cells to FK866+*β*-lap results in synergistic depletion of NAD^+^/NADH, NADP^+^/NADPH, glutathione, and ATP pools. (**a** and **b**) MiaPaca2 cells were pretreated with FK866 (8 nM, 24 h), with or without NMN (500 *μ*M) and then exposed or not to various doses of *β*-lap (*μ*M, 2 h with or without FK866 ± NMN). Total NADH & NAD^+^ (**a**) or NAD(*P*)H & NAD(*P*)^+^ (**b**) pools were assayed at 2 h when drugs were removed. NMN addition partially rescued depletion of both sets of nucleotides. Results were analyzed by ANOVA (*n*=3 ± S.D.) comparing *β*-lap alone (no addition) with FK866+*β*-lap. (**c**) MiaPaca2 cells were treated as in **a** and **b** and relative reduced glutathione (GSH) levels (T/C) were monitored. (**d**) MiaPaca2 cells were pretreated as in **a** and **b** with FK866 (8 nM) and then with or without *β*-lap (2.5 *μ*M) in the presence or absence of FK866 (8 nM) and relative ATP pools were assessed. Student's *t*-tests were performed (*n*=3 ± S.D.). *P<0.05; **P<0.01; ****P<0.0001

*β*-Lap-treated cells exhibit severe NQO1-dependent NAD^+^/NADH depletion at lethal doses as a result of a two-step process: (i) NQO1-dependent conversion of NADH to elevated pools of NAD^+^ with accompanying elevation of ROS levels; and (ii) rapid depletion of NAD^+^ levels owing to ROS-stimulated PARP1 hyperactivation.^9, 13, 16, 32, 33^ When MiaPaca2 cells were exposed to FK866+*β*-lap, a significantly greater NAD^+^/NADH depletion was noted as a result of the smaller NAD+ pool size caused by the inhibition of NAMPT-catalyzed nicotinamide recycling (Figure 3a), with similar findings in other PDA cell lines. Although low doses of *β*-lap (1–2 *μ*M) cause PARP1 activation and NAD^+^ consumption, NAD^+^ synthesis compensates to maintain the pool size. However, when NAD^+^ synthesis was inhibited by FK866, the shoulder region where NAD^+^/NADH levels were maintained with low doses of *β*-lap was eliminated (Figure 3a). Addition of NMN spared, to some extent, loss of NAD^+^/NADH total pools caused by FK866+*β*-lap (Figure 3a). Though complete rescue of nucleotide pools with NMN did not occur, this partial rescue is relevant as it prevented reduction in cell viability from the effects of combination treatment (Figure 2c). NADP^+^/NADPH losses followed similar trends, but with less overall depletion (Figure 3b), most likely because NADP^+^ is not directly consumed by PARP1, but is synthesized from NAD^+^.

As reduced glutathione (GSH) is oxidized to neutralize ROS generated by *β*-lap, we explored the effects FK866+*β*-lap combination therapy on GSH. We noted significant depletion of GSH levels in *β*-lap-exposed MiaPaca2 cells, which was lower after combination treatment and rescued with NMN (Figure 3c).^34^ Additional loss of GSH levels could be a result both of decreased glutathione reductase activity due to low NADPH levels (Figure 3b) and less *de novo* GSH synthesis caused by ATP loss noted with *β*-lap.^29, 35^ Indeed, additional ATP loss was noted in MiaPaca2 cells exposed to FK866 (8 nM)+*β*-lap (2.5 *μ*M) compared with either agent alone (Figure 3d).

### Synergistic inhibition of glycolysis by FK866+*β*-lap

**Figure 4:**
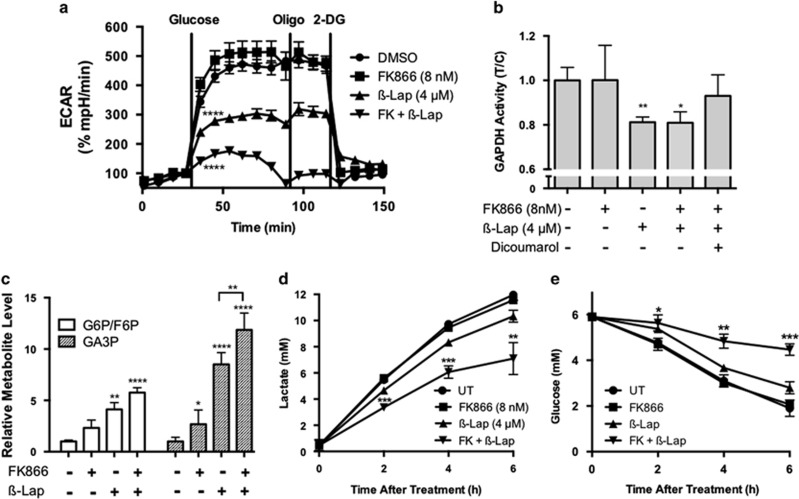
FK866+*β*-lap treatment results in synergistic inhibition of glycolysis. (**a**) Glycolytic stress tests were performed using the Seahorse XF bioanalyzer to measure the glycolytic capacity of MiaPaca2 cells pretreated with or without FK866 (8 nM), *β*-lap (4 *μ*M) alone, or the combination therapy of FK866 (8 nM)+*β*-lap (4 *μ*M). Analyses were performed immediately following 2 h *β*-lap exposures. Results were compared using ANOVA (*n*=4). ****P<0.0001. (**b**) GAPDH enzyme activities were measured from lysates of MiaPaca2 cells with or without FK866 (8 nM), and then exposed or not to *β*-lap in the presence or absence of dicoumarol (50 *μ*M) for 2 h. Extracts were prepared and enzyme activities (*μ*mol NADH/min) were monitored and normalized to untreated samples. Data were expressed as relative GAPDH activity (T/C) and results were compared using Student's *t*-tests (*n*=6, ± S.D.) *P<0.05; **P<0.01. (**c**) Glucose 6-phosphate/fructose 6-phosphate (G6P/F6P) and glyceraldehyde 3-phosphate (GA3P) were quantified in MiaPaca2 cells 30 min after treatment as described in methods. (**d** and **e**) MiaPaca2 cells were treated as in **a** and extracellular lactate (**d**) or glucose (**e**) levels in culture media were quantified using a Nova Bioprofile Analyzer at the indicated times after both removal of drugs. Results were compared using Student's *t*-tests (*n*=3 ± S.D.) *P<0.05;**P<0.01; ***P<0.001;****P<0.0001

As marked alterations in ATP levels were noted with combination treatment, we assayed MiaPaca2 cells for glycolytic activity after *β*-lap+FK866 treatment. Analysis of extracellular acidification rate (ECAR) with the XF Flux Analyzer revealed that basal glycolysis and glycolytic reserve capacity of *β*-lap-exposed cells were reduced immediately after drug exposure (Figure 4a). Prior studies have shown that long-term FK866 treatment inhibited glycolysis by reducing glyceraldehyde 3-phosphate dehydrogenase (GAPDH) activity owing to low NAD^+^ levels.^36, 37^ We found that short-term (24 h) treatment with FK866 followed by removal of the drug resulted in no ECAR reduction (Figure 4a). Nevertheless, FK866 pretreatment drastically decreased ECAR following *β*-lap co-treatment. As GAPDH can be reversibly inhibited by ROS-induced oxidation or irreversibly by post-translational modifications (e.g., PARylation),^38, 39, 40, 41^ we studied whether *β*-lap affected GAPDH activity. Indeed, GAPDH activity was inhibited in MiaPaca2 cells following *β*-lap exposure (Figure 4b). Excess NADH was added to the assay buffer, so the moderate GAPDH inhibition seen in Figure 4b is independent of NAD^+^ depletion.^36^ Importantly, FK866 pretreatment did not reduce GAPDH inhibition caused by *β*-lap exposure, but Dic prevented this inhibition (Figure 4b). These data suggest that *β*-lap causes ROS-induced stable post-translational modification of GAPDH, whereas increased glycolytic inhibition observed with combination treatment was secondary to increased NAD^+^ depletion (Figures 3a and 4a and b). Inhibition of GAPDH is strongly supported by a ~12-fold accumulation of glyceraldehyde 3-phosphate (GA3P), the substrate of GAPDH, and a lesser accumulation of the upstream metabolites glucose-6-phosphate/fructose-6-phosphate (G6P/F6P) in *β*-lap+FK866 treated cells (Figure 4c). Additional accumulation of these metabolites in *β*-Lap+FK866 treated *versus* *β*-lap alone treated MiaPaca2 cells (*P*<0.01) was consistent with the reduced ECAR after combination treatment owing to lower NAD^+^ pools despite the similar level of NAD^+^-independent enzyme inhibition observed in Figure 4b. Next, we explored whether the inhibition of glycolysis with FK866+*β*-lap treatment was durable. Synergistic inhibition of lactate production and glucose utilization was noted following drug removal after combination treatment, but before cells begin to die (at 12–48 h), suggesting that the combined effects of *β*-lap on GAPDH inhibition and both drugs on NAD^+^ depletion caused long-term inhibition of glycolysis in PDA cells (Figures 4d and e).

### FK866+*β*-lap combination therapy results in significantly lowered PAR formation

**Figure 5:**
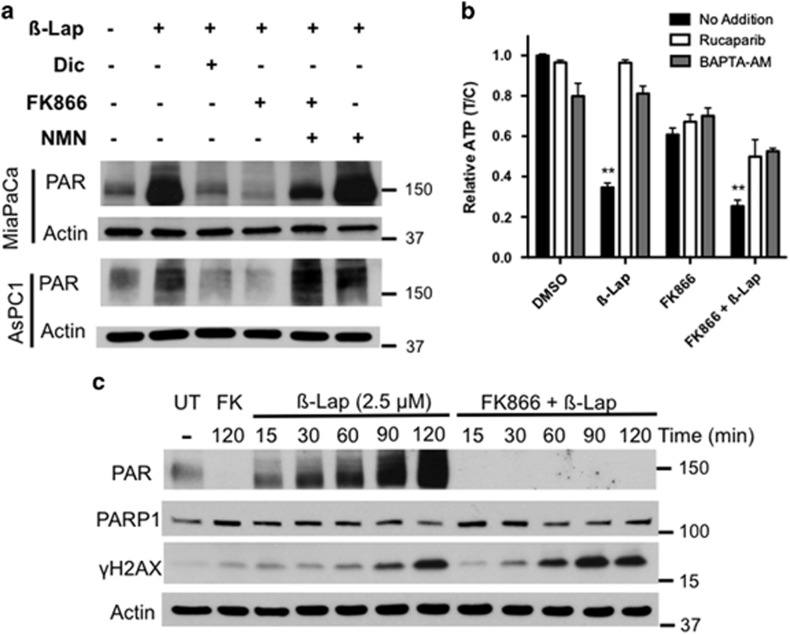
FK866 addition abrogated PAR formation and increased DSBs in *β*-lap-treated MiaPaca2 cells. (**a**) NQO1+ MiaPaca2 or AsPC1 PDA cells were pretreated with FK866 (8 nM, 24 h), then *β*-lap (2.5 *μ*M) was added in the presence or absence of dicoumarol (Dic, 50 *μ*M) or NMN (500 *μ*M) for 20 min. Cell extracts were analyzed for PAR formation. Dicoumarol inhibits NQO1 activity thereby preventing PARP1 hyperactivation. *β*-Lap-induced PAR formation was abrogated by FK866 (8 nM) and rescued with NMN (500 *μ*M). Actin levels were monitored as loading controls. (**b**) MiaPaca2 cells were pretreated with or without FK866 (8 nM) and then exposed to *β*-lap (3 *μ*M) in the presence or absence of (i) a PARP1 inhibitor (Rucaparib (AG014699), 20 *μ*M) or (ii) a calcium chelator (BAPTA-AM) for 2 h; Rucaparib or BATPA-AM treatments prevent PARP1 hyperactivation in *β*-lap-treated cells.^16, 29, 42^ ATP levels were measured after the 2 h co-treatment. Student's *t*-tests were performed comparing *β*-lap or FK866+*β*-lap *versus* addition of Rucaparib (AG014699) (*n*=3 ± S.D.). **P <0.01. (**c**) MiaPaca2 cells were pretreated or not with FK866 (8 nM, 24 h), then exposed to *β*-lap (2.5 *μ*M) and PAR and *γ*-H2AX formation were monitored at various times during the 2 h exposure by western blot. Mean *γ*H2AX band intensities, as a measure of DNA double-strand break formation, were normalized to actin and levels graphed over time (mins) in Supplementary Figure S1D. Reduced PARP1 activity, as a result of lowered NAD+ levels from FK866 exposure, resulted in more rapid induction of *γ*-H2AX

We previously reported that *β*-lap-induced lethality was caused by PARP1 hyperactivation and marked loss of NAD^+^ pools in NQO1+ cancer cells, including PDA.^13^ As others have shown that the PAR polymer itself is critical for signaling and lethality in other forms (e.g., MNNG exposure) of PARP1 hyperactivation-mediated cell death, we sought to define the distinct contributions of PARP1 activity and PAR formation in the synergy noted with FK866+*β*-lap combination treatment.^26^ *β*-Lap and FK866 combination treatment of NQO1+ MiaPaca2 or AsPC1 cells resulted in virtually undetectable PAR formation by western blot, in contrast to the elevated PAR levels typically noted in *β*-lap-treated NQO1+ cancer cells (Figure 5a). This effect was rescued by restoring NAD^+^ levels with NMN. Despite decreased NAD^+^ pools caused by FK866 pretreatment, exposure of *β*-lap-treated MiaPaca2 cells to the PARP1 inhibitor, Rucaparib (AG014699), or addition of BAPTA-AM to chelate Ca^2+^ (which is required for *β*-lap-induced PARP1 hyperactivation),^29, 42^ prevented additional ATP depletion caused by *β*-lap treatment in combination with FK866 (Figure 5b). These findings strongly suggest that reduction of NAD^+^ pools by FK866 treatment increases the ability of PARP1 to completely exhaust NAD^+^ pools after *β*-lap treatment, without generating substantial PAR formation (Figures 5b and c). Once NAD^+^ levels are exhausted, PARP1 activity would not function properly to repair DNA lesions created by the high levels of ROS induced by *β*-lap, resulting in more rapid conversion to DNA double-strand breaks. Indeed, delayed γH2AX induction normally seen with *β*-lap treatment alone appeared more rapidly in MiaPaca2 cells exposed to FK866+*β*-lap (Figure 5c,Supplementary Figure S1E).

### ROS formation and mechanism of cell death

**Figure 6:**
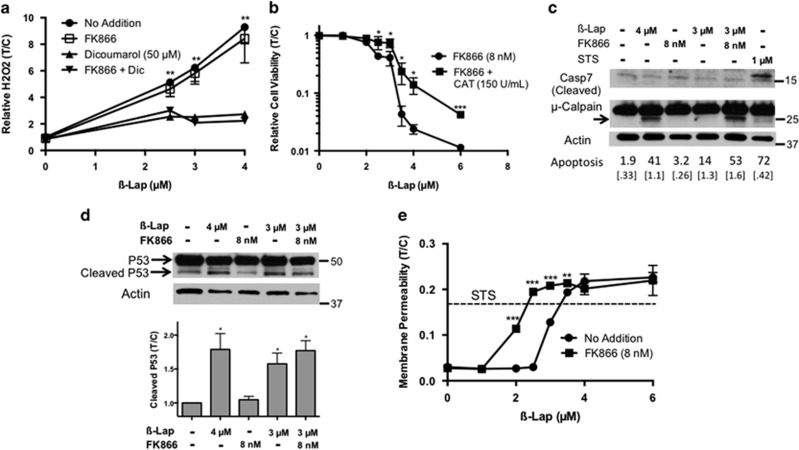
Exposure of NQO1+ MiaPaca2 cells to FK866+*β*-lap leads to NQO1-dependent, *μ*-calpain-mediated cell death. (**a**) H_2_O_2_ levels, resulting from NQO1-dependent futile cycling of *β*-lap, were monitored in MiaPaca2 cells treated as in 5**a** using CellRox-Glo, normalized to DMSO-treated cells. FK866 pre- and co-treatment with *β*-lap (*μ*M) did not alter H_2_O_2_ production over the 2 h treatments. (**b**) Addition of pegylated catalase (150 U/ml) neutralized H_2_O_2_ formation in FK866 (8 nM)+*β*-lap (*μ*M)-exposed MiaPaca2 cells over a 2 h treatment period, which partially rescued combination treatment lethality. (**c**) Proteolytic cleavage of caspase-7 and the small subunit (27 kDa, arrow) of *μ*-calpain as indicators of activation were monitored by western blot 48 h post-treatment in MiaPaca2 cells pretreated with FK866 (8 nM), with or without *β*-lap (4 *μ*M) for 2 h. Staurosporin (1 *μ*M, 18 h) treatment of MiaPaca2 cells served as a positive control for classic apoptosis. Flow cytometry analysis of subG_0_G_1_ cells after the same treatment conditions was performed (S.D. in brackets), histogram in Supplementary Figure S2A. (**d**) Atypical p53 proteolysis, as expected with *μ*-calpain-induced cell death, was observed 48 h after exposure to *β*-lap alone or after combination FK866+*β*-lap treatment. Band intensity was quantified and normalized to actin band intensity as indirect quantification of μ-calpain activity. (**e**) MiaPaca2 cells were pre- and co-treated with or without FK866 (8 nM, 24 h) and then exposed or not to FK866+*β*-lap (*μ*M) for 2 h. Membrane permeability was measured 24 h after drug treatment using CellTox Green, a membrane impermeable dye that measures extracellular DNA. Cells were lysed with SDS for 100% permeability normalization. For comparison, STS induced intrinsic apoptosis, but not necrosis. All indicated results were compared with Student's *t*-tests (*n*=3, ± S.D.). *P<0.05; **P<0.01; ***P<0.001

One concern in lowering NAD^+^/NADH levels is that the NQO1-dependent futile cycle could be compromised. However, we noted that H_2_O_2_ levels generated in MiaPaca2 cells after *β*-lap treatments with or without FK866 (8 nM, pre- and co-administration) were identical (Figure 6a). Furthermore, addition of pegylated catalase (150 U/ml) partially rescued lethality in MiaPaca2 cells exposed to *β*-lap+FK866 (Figure 6b) in a manner similar to rescue reported in cells treated with *β*-lap alone.^13, 43^

We hypothesized that MiaPaca2 cells exposed to FK866+*β*-lap would die by the same caspase-independent, but apoptosis-like programmed cell death pathway that occurs in *β*-lap-treated NQO1+ cancer cells.^30^ Exposing MiaPaca2 cells to 4 *μ*M *β*-lap for 2 h resulted in significant apoptosis (41%); cells lacked caspase cleavage,^9^ and demonstrated *μ*-calpain activation by the formation of the 23 kDa active form of the small subunit of *μ*-calpain 48 h post-treatment (Figure 6c). Atypical cleavage of p53 catalyzed by *μ*-calpain was also observed in *β*-lap and combination treated cells (Figure 6d).^12^ Exposing MiaPaca2 cells to FK866+*β*-lap resulted in significant levels of apoptotic cells (>50%) that appear to die by the same mechanism (Figures 6c and d) as noted with a lethal dose of *β*-lap.^9, 16^ Treatment of MiaPaca2 cells with FK866 (8 nM, 24 h) alone did not cause substantial apoptosis (Figure 6c). As PAR accumulation was not noted in the cell death induced by FK866+*β*-lap, we conclude that free PAR is not necessary in this NAD^+^ loss-dependent cell death pathway (Figures 5 and 6).

Depletion of NAD+/NADH, NADP+/NADPH, and ATP occurred to a greater extent with *β*-lap+FK866 combination treatment than with *β*-lap alone (Figure 3), which could promote necrosis, so we examined changes in cell membrane permeability. We found that membrane permeability after exposure to FK866+*β*-lap did not surpass that of cells treated with *β*-lap alone, and that both were comparable to cells undergoing canonical apoptosis induced by the apoptosis-inducing agent, staurosporine (Figure 6e) The rounded, condensed nuclei and reduction of cytoplasm observed after combination treatment is also similar to the morphology of apoptotic cells (Supplementary Figure S2B).

## Discussion

Addition of *β*-lap to short-term FK866 treatment resulted in enhanced tumor-cell specificity and efficacy, not typically found with FK866 treatment alone. This combination resulted in elevated DNA lesions, more extensive NAD^+^/ATP loss mediated by PARP1, and metabolic catastrophe. MNNG, an alkylating agent that causes DNA damage and PARP1 hyperactivation, was reported to block glycolysis through a free PAR moiety-mediated mechanism of hexokinase inhibition.^27^ In contrast, *β*-lap exposure caused the inhibition of GAPDH even in the presence of excess NADH, which could be a result of oxidative stress- or PARylation-induced post-translational modification.^40^ However, PAR did not accumulate in cells co-treated with FK886+*β*-lap, yet persistent GAPDH inhibition was noted to the same extent as cells exposed to *β*-lap alone. Although the mechanism by which GAPDH is inhibited by combination treatment is not definitively known, there are a few probable options. We previously reported NQO1-dependent, ROS-induced S-nitrosylation and nuclear translocation of GAPDH in various NQO1 cancer cells after *β*-lap treatment,^13^ and reactive nitrogen species-mediated irreversible inhibition of GAPDH has also been previously shown *in vitro*.^44^ Regardless of the specific mechanism, the inhibition of GAPDH by *β*-lap was enhanced by the additional NAD^+^ depletion caused by combination treatment with FK866, as seen by drastically increased accumulation of GA3P, and has long-term effects on glycolytic activity. In addition, our data strongly suggest that free PAR accumulation is not a major component. Without active ATP synthesis, cancer cells cannot recover from depleted NAD^+^ pools or repair the extensive DNA lesions caused by *β*-lap, resulting in cell death.^29^

Addition of FK866+*β*-lap to NQO1-overexpressing cancer cells caused a burst of ROS-induced DNA damage and NAD^+^/ATP depletion in a near-identical manner to the caspase-independent, *μ*-calpain-mediated cell death pathway induced by lethal doses of *β*-lap alone, despite the additional energy depletion. Death occured in over 90% of asynchronous cancer cells after 2 h of combination treatment, and the cell cycle distribution was not altered post treatment, suggesting cell cycle independence. There are reports of *β*-lap inducing caspase-mediated apoptosis, but this is noted only after long-term continuous treatment with *β*-lap, which also reduces specificity for NQO1+ overexpressing cells.^45, 46, 47^ Others have reported that PAR polymers directly influence *μ*-calpain activation, and are critical for inducing cell death under conditions where PARP1 hyperactivation occurs, such as with MNNG treatment or ischemia-reperfusion.^25, 26^ With the treatment conditions reported herein, PARP1 activity is stimulated by *β*-lap, but extensive PAR formation did not occur owing to low NAD^+^ levels, and rescuing PAR formation and NAD^+^ levels with NMN decreases cytotoxicity. Nevertheless, *μ*-calpain activation/proteolysis and caspase-independent apoptosis still occured, demonstrating that PARP1 hyperactivation to deplete energy stores was more important than the formation and accumulation of extensive PAR polymers. Our findings with *β*-lap are consistent with a report that NAMPT inhibition synergizes with pemetrexed in a PARP1-dependent manner with an associated reduction in PAR formation.^48^ We find that severe NAD^+^/NADH depletion induced by *β*-lap causes metabolic catastrophe, ATP depletion, and cell death that is independent of PAR accumulation and is promoted by NAMPT inhibition. We refer to this mode of cell death as NAD^+^-Keresis, after the death spirits in Greek mythology called Keres, who pull the life out of those who die violently, in this case through severe NAD^+^/NADH depletion.

Novel therapies are desperately needed for patients with PDA. By combining NAMPT inhibitors (e.g., FK866) and NQO1 bioactivatable drugs (e.g., *β*-lap, ARQ761) we exploit the reliance of PDA on rapid NAD^+^ synthesis, as well as the tumor-selective overexpression of NQO1 through the use of agents that are bioactivated to induce cell death. Combination treatment with NAMPT inhibitors+*β*-lap addresses issues associated with either agent alone. This combination is expected to enhance efficacy at well-tolerated doses of these drugs. We noted that FK866+*β*-lap combination treatment was most effective with FK866 pretreatments followed by *β*-lap co-treatment. This was consistent with our hypothesis that lowered NAD^+^/NADH levels caused by FK866 would sensitize NQO1+ tumor cells to rapid futile redox cycling initiated by *β*-lap. Importantly, cytotoxicity of these drugs to normal cells is not increased with combination treatment; elevated NQO1 expression is still required to achieve cell death. Furthermore, the *β*-lap doses used in our studies are relevant to those achievable *in vivo.*^49^ Likewise, the maximum tolerated dose of FK866 in clinical trials yielded a 14 nM steady state plasma concentration, which is in the effective synergy range for combination treatment with *β*-lap.^50^ However, for single-agent treatment, it was administered continuously for 96 h. Combination treatment with *β*-lap significantly reduced the required FK866 treatment time, which should increase the feasibility of its clinical application. Next-generation NAMPT inhibitors currently in development, which demonstrate better PK profiles, are expected to similarly benefit from combination treatment, with reduced need for long-term infusion.^51^ This treatment strategy will be pursued in further preclinical studies to explore potential clinical application and to further elucidate the pathways and mechanism of NAD-Keresis.

## Materials and Methods

### Chemicals and reagents

We synthesized and purified *β*-lap and prepared a stock solution at 50 mM in DMSO. FK866 hydrochloride hydrate, GMX1778, Dic, catalase-polyethylene glycol, and BAPTA-AM were purchased from Sigma-Aldrich (St. Louis, MO, USA).

### Cell culture

Cell lines were obtained from ATCC. They were grown in DMEM (Life Technologies, Carlsbad, CA, USA) containing 10% FBS (Fisher Scientific, Waltham, MA, USA) in a 37 ° incubator with 5% CO_2_. Cells were tested monthly to confirm the absence of mycoplasma contamination. Lipofectamine RNAiMAX (Life Technologies) was used for siRNA transfections. Cells were transfected with one of two siRNAs purchased from Sigma-Aldrich to target NAMPT (NAMPT #1: SASI_Hs02_00340191, NAMPT #2: SASI_Hs02_00340192, or a non-targeting control siRNA). After 48 h of incubation with RNAiMax and siRNA in OptiMEM (Life Technologies), cells were detached with trypsin/EDTA (Life Technologies) and seeded for treatment assays or lysed for analysis of knockdown efficiency. For combination drug treatments, cells were treated for 24 h with FK866 followed by co-treatment with *β*-lap in complete media. Two hours after *β*-lap addition, drug-containing media were removed and replaced with fresh media.

### Clonogenicity

After *β*-lap, FK866, or combination treatment at 60% confluency, cells were trypsinized, counted with a Coulter Counter, and diluted in single-cell suspension. Cells were then seeded at 100, 500, or 1000 cells per plate on 60 mm plates and allowed to proliferate for 7 days. Plates were washed in PBS and cells were fixed and dyed with methanol/crystal violet. Colonies of 50 or more cells with normal appearance were counted and results were normalized to the colonies formed without drug treatment.

### Nucleotide, GSH, and H2O2 assays

CellTiter-Glo (Promega, Madison, WI, USA) was used for cell viability assays (24 h after treatment) and ATP assays (at indicated time points during or after *β*-lap treatment). The following assays were purchased from Promega: GSH/GSSG-Glo, NAD/NADH-Glo, NADP/NADPH-Glo, and ROS-Glo and were used as directed. Unless otherwise noted, all raw luminescent values for treatment conditions were normalized to the signal from untreated cells (T/C). Standard curves were generated to ensure linearity.

### Immunoblotting

Cells were lysed in ice-cold RIPA with protease and phosphatase inhibitors (Santa Cruz, Dallas, TX, USA). Whole-cell extracts were prepared by centrifugation at 14 000 × *g* to remove insoluble components. Protein concentration was determined by BCA assay (Thermo Scientific, Waltham, MA, USA) and loading volume was normalized. Extracts were run on 8 or 4–20% (Bio-Rad, Hercules, CA, USA) gradient acrylamide SDS-PAGE gels and transferred to PVDF membrane. Primary antibodies for protein detection included: phospho-H2A.X (JBW301, Millipore, Billerica, MA, USA), PARP1 (F-2, Santa Cruz), PAR (Trevigen, Gaithersburg, MD, USA), Actin (C-2, Santa Cruz), visfatin/NAMPT (rabbit polyclonal, Abcam, Cambridge, UK), small subunit calpain (EPR3324, Abcam). Primary hybridization was carried out in Sigma casein blocking buffer at 4 ° overnight. Secondary HRP conjugated antibodies were incubated for 1 h at room temperature, followed by detection with SuperSignal West Pico (Thermo Scientific). Bands were quantified by mean intensity in ImageJ and normalized to the actin band intensity to control for loading variation.

### Glycolytic flux

A Seahorse XF24 bioanalyzer (Seahorse Bioscience, North Billerica, MA, USA) was used for glycolytic stress tests. Cells were seeded at 3 × 10^4^ cells/well in 24-well plates and were treated with *β*-lap at 4 *μ*M for 2 h in complete media and washed with fresh Seahorse media. The glycolytic stress test kit was used to inject glucose, oligomycin, and 2-deoxy-D-glyucose at the indicated times.

### GAPDH activity

Cells were pretreated ± FK866 for 24 h, co-treated ± *β*-lap for 2 h, washed with PBS, and assayed for GAPDH activity using the KDalert GAPDH activity assay (Life Technologies) as directed.

### Metabolomics

Subconfluent MiaPaca2 cells were pretreated ± FK866 for 24 h and co-treated with *β*-lap for 30 min. Cells were washed twice with ice-cold saline, then scraped in methanol/water (50/50, v/v). Cells were subjected to three freeze–thaw cycles. After rigorous vortexing, cell debris was removed by centrifugation. Pellets were used for protein quantitation (BCA Protein Assay, Thermo Scientific). The supernatant was evaporated to dryness using a SpeedVac concentrator (Thermo Savant, Holbrook, NY, USA) and metabolites were reconstituted in 0.03% formic acid in analytical-grade water and centrifuged to remove insoluble debris. Supernatants were transferred to HPLC vials for metabolomics analyses.

Targeted metabolite profiling was performed using a liquid chromatography-mass spectrometry/mass spectrometry approach. Separation was achieved on a Phenomenex Synergi Polar-RP HPLC column (150 × 2 mm, 4 *μ*M, 80 Å) using a Nexera Ultra High Performance Liquid Chromatograph system (Shimadzu Corporation, Kyoto, Japan). The mobile phases used were 0.03% formic acid in water (A) and 0.03% formic acid in acetonitrile (B). The gradient program was as follows: 0–3 min, 100% A; 3–15 min, 100–0% A; 15–21 min, 0% A; 21–21.1 min, 0–100% A; 21.1–30 min, 100% A. The column was maintained at 35 °C and samples were kept in the autosampler at 4 °C. The flow rate was 0.5 ml/min, and injection volume 10 *μ*l. The mass spectrometer was an AB QTRAP 5500 (Applied Biosystems SCIEX, Foster City, CA) with electrospray ionization source in multiple reaction monitoring (MRM) mode. Sample analyses were performed in positive/negative switching mode. Declustering potential and collision energy were optimized for each metabolite by direct infusion of reference standards using a syringe pump prior to sample analysis. The MRM MS/MS detector conditions were set as follows: curtain gas 30 psi; ion spray voltages 5000 V (positive) and −1500 V (negative); temperature 650 °C; ion source gas 1 50 psi; ion source gas 2 50 psi; interface heater on; entrance potential 10 V. Dwell time for each transition was set at 3 msec. MRM data were acquired using Analyst 1.6.1 software (Applied Biosystems SCIEX). Chromatogram review and peak area integration were performed using MultiQuant software version 2.1 (Applied Biosystems SCIEX). The integrated peak area values were used as variables for the statistical data analysis. The chromatographically co-eluted metabolites with shared MRM transitions were shown in a grouped format, that is, G6P/F6P.

### Lactate and glucose quantification

Cells were pretreated with FK866 for 24 h and co-treated with *β*-lap for 2 h in complete media. After co-treatment, media was replaced with low glucose, phenol-free DMEM (Invitrogen) with 5% FBS and collected at indicated times for analysis with a BioProfile Automated Analyzer (Nova Biomedical, MA, USA).

### Flow cytometry

For cell cycle analysis, cells were pretreated with FK866 followed by co-treatment with *β*-lap for 2 h. Drug-containing media was removed and cells were incubated in fresh complete media for 48 h. Cells were trypsinized, and both adherent and floating cells were collected and washed in 1% BSA in PBS. After fixing cells in 70% ethanol, cells were washed and resuspended in BSA/PBS buffer containing propidium iodine and saponin. Cells were analyzed on a FACSAria (BD Biosciences, San Jose, CA, USA) and cell cycle distribution was calculated in FlowJo.

### Statistics

Unless otherwise noted, graphs are plotted as mean with error bars denoting S.D. Curve fitting and calculation of IC50 values, ANOVA, and two-tailed student *t*-tests for statistical significance with Holm/Sidak multiple comparison correction were performed in GraphPad Prism 6.

## Abbreviations

PDA: pancreatic ductal adenocarcinoma
NAD: nicotinamide adenine dinucleotide
ADP: adenosine diphosphate
PAR: poly(ADP ribose)
NAMPT: nicotinamide phosphoribosyltransferase
NQO1: NADPH:quinone oxidoreductase
PARP1: poly(ADP ribose) polymerase
AIF: apoptosis-inducing factor
GAPDH: glyceraldehyde 3-phosphate dehydrogenase
*β*-lap: *β*-lapachone
ROS: reactive oxygen species
MNNG: methylnitronitrosoguanidine
ATP: adenosine triphosphate
DER: dose enhancement ratio
Dic: dicoumarol
NMN: nicotinamide mononucleotide
GSH: glutathione (reduced)
ECAR: extracellular acidification rate
Oligo: oligomycin
2-DG: 2-deoxy-D-glucose
FK: FK866
G6P: glucose 6-phosphate
F6P: fructose 6-phosphate
GA3P: glyceraldehyde 3-phosphate
STS: staurosporine
RNS: reactive nitrogen species
PK: pharmacokinetics
