# Supplementary figures and images for: NAMPT inhibition sensitizes pancreatic adenocarcinoma cells to tumor-selective, PAR-independent metabolic catastrophe and cell death induced by β-lapachone
Source: Cell Death Dis. 2015 Jan 15;6(1):e1599–. doi: 10.1038/cddis.2014.564 (PMC4669762; doi:10.1038/cddis.2014.564)

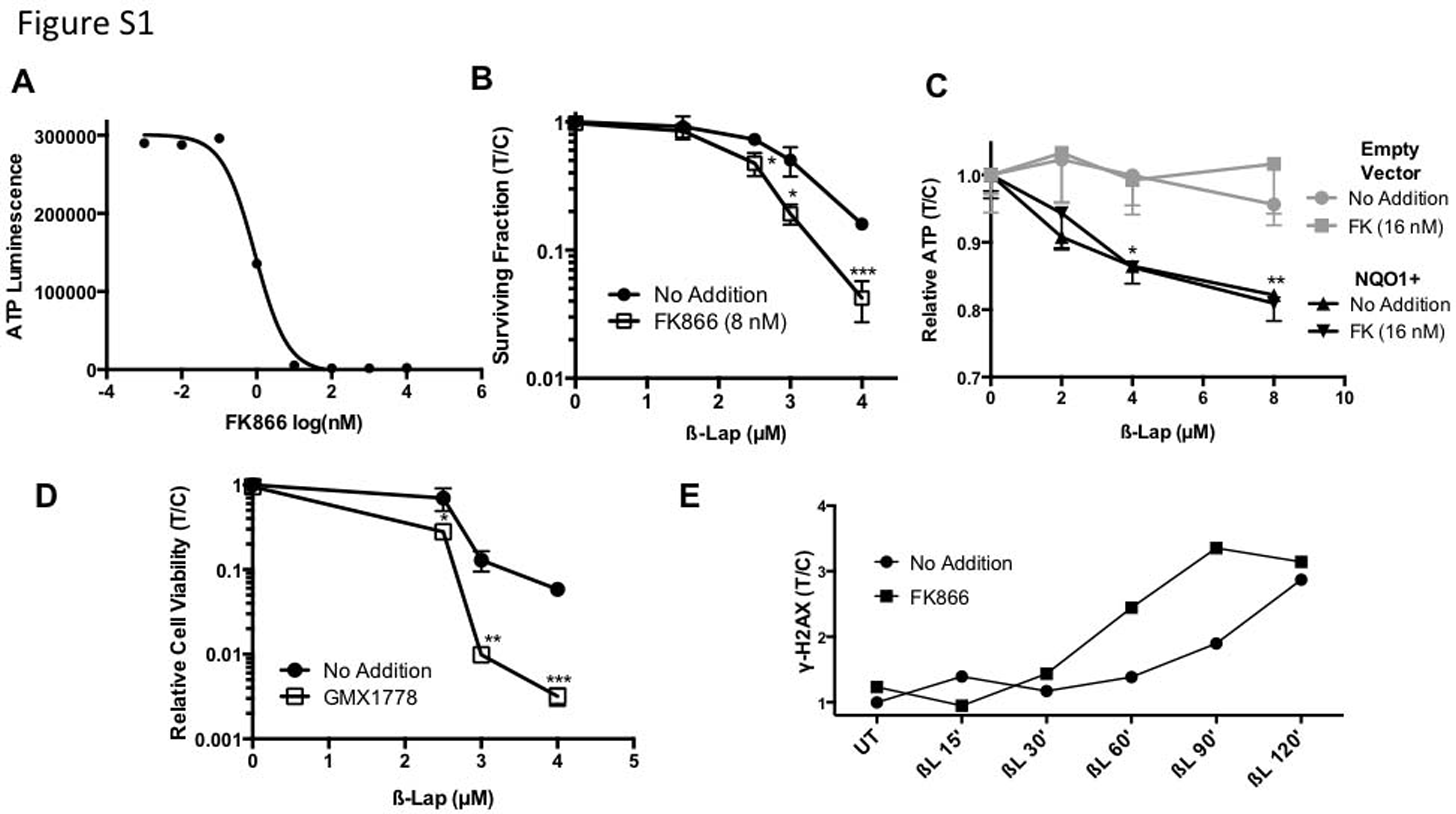

Supplement: Supplementary Figure S1 [file cddis2014564x1.tif]

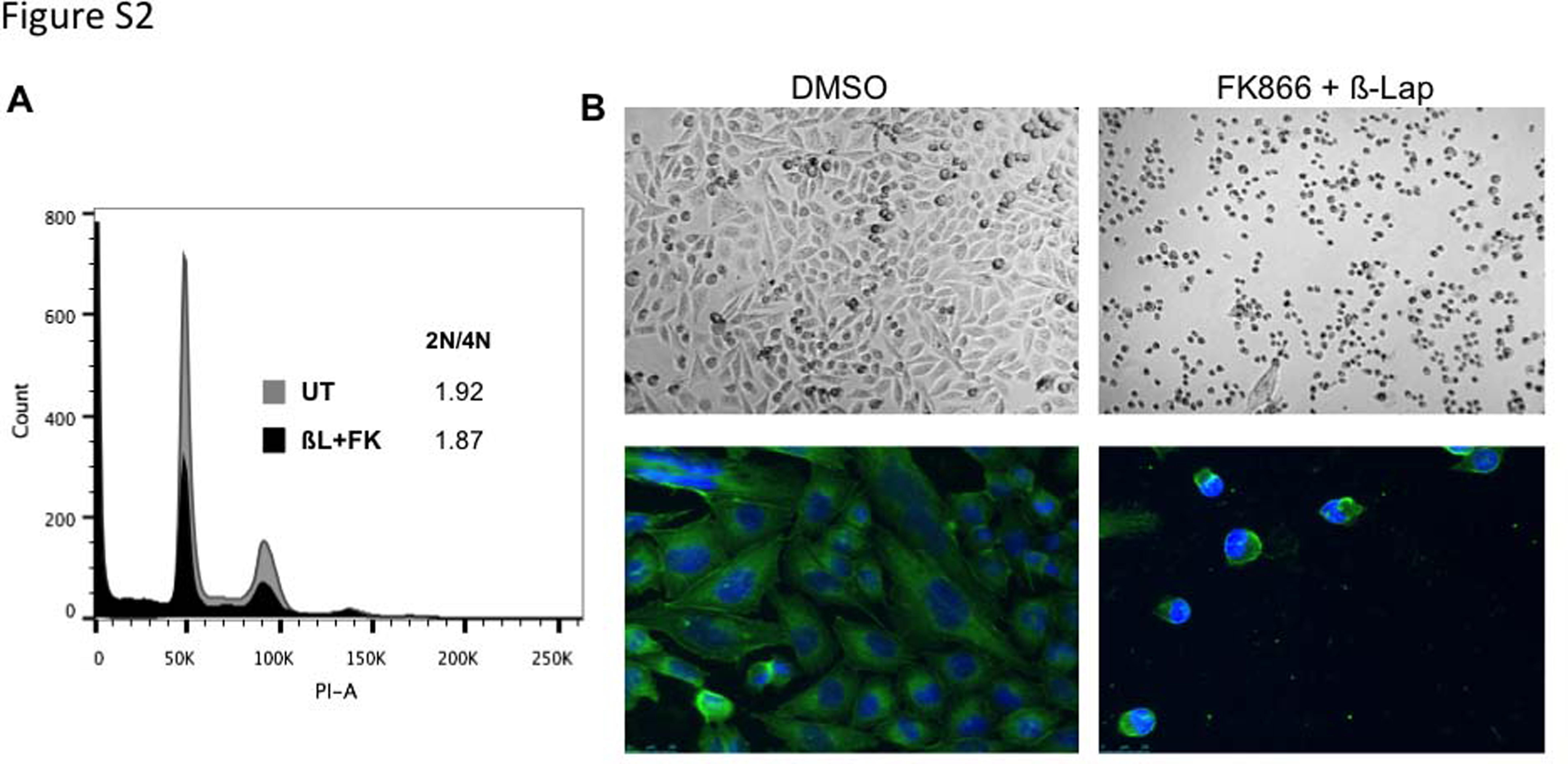

Supplement: Supplementary Figure S2 [file cddis2014564x2.tif]
