# Supplementary material for: NAMPT inhibition sensitizes pancreatic adenocarcinoma cells to tumor-selective, PAR-independent metabolic catastrophe and cell death induced by β-lapachone
Source: Cell Death Dis. 2015 Jan 15;6(1):e1599–. doi: 10.1038/cddis.2014.564 (PMC4669762; doi:10.1038/cddis.2014.564)
Supplement: Supplementary Figure Legends [file cddis2014564x3.doc]

**Figure S1: Alternative combination treatment conditions and γH2AX quantification. S1A**) Sensitivity of MiaPaca2 cells, as monitored by loss of ATP, to long-term (72 h) FK866 treatments. **S1B**) Cells pretreated 24 h +/- 8 nM FK866 and co-treated with ß-lap were assayed for clonogenic survival using colony forming assays (CFAs). Colonies with >50 normal-appearing cells were counted and normalized to the colony numbers from untreated control cells. **S1C**) IMR90 fibroblasts were transfected with NQO1 or empty expression vectors. Cells were treated 24 h +/- 16 nM FK866 and then co-treated with ß-lap for 2 h. ATP levels were assayed after combination treatment. **S1D**) Synergy between ß-lap and GMX1778 (64 nM) combination treatment. **S1E**) Quantification of γH2AX formation by Western blot as a marker of DNA double strand break (DSB) formation from Fig. 5C. Raw band intensities were normalized to actin band intensities from the same lanes and normalized to background intensities from untreated MiaPaca2 cells.

**Figure S2: Death of cancer cells after combination treatment. S2A**) DNA content histogram of MiaPaca2 cells 48 h after FK866 + ß-lap treatment exhibits cell cycle distribution ratio similar to untreated cells with the exception of a large subG_0_/G_1_ population. **S2B**) Phase contrast (above, 10x) and immunofluorescence (below, 40x, green: actin, blue: DAPI) of vehicle treated and FK866 (8 nM) + ß-lap (4 µM) treated MiaPaca2 cells shows rounded, condensed nucleus and reduction of cytoplasm 24 h after combination treatment.
